# Impact of the COVID-19 Pandemic on Emergency Care Utilization and Outcomes in Pediatric Patients with Intussusception

**DOI:** 10.3390/children9020277

**Published:** 2022-02-17

**Authors:** Jin Hee Lee, Young Sun Ro, Hyuksool Kwon, Dongbum Suh, Sungwoo Moon

**Affiliations:** 1 Department of Emergency Medicine, Seoul National University Bundang Hospital, Seongnam 13620, Korea; gienee@snubh.org (J.H.L.); jinuking3g@naver.com (H.K.); dongbums@gmail.com (D.S.); 2 Department of Emergency Medicine, Seoul National University College of Medicine, Seoul 03080, Korea; 3 Department of Emergency Medicine, Seoul National University Hospital, Seoul 03080, Korea; 4 National Emergency Medical Center, National Medical Center, Seoul 04564, Korea; sungwoo.moon89@gmail.com; 5 Department of Emergency Medicine, Korea University Ansan Hospital, Ansan 15355, Korea

**Keywords:** COVID-19, intussusception, pediatric

## Abstract

Background: We determined whether a decrease in healthcare utilization patterns during the COVID-19 pandemic affected the treatment process of pediatric patients with intussusception. Methods: Patients with suspected intussusception who had ICD-10 code K561 as their discharge diagnosis from the national database were selected, and those who underwent either radiologic and/or surgical reduction were defined as true intussusception patients. We compared the time periods from patients visiting the ED to ultrasound, radiologic reduction and/or surgical reduction between the study group (first half of 2020, COVID-19 period) and the control groups (control group 1: first half of 2019, control group 2: second half of 2019). Results: The number of suspected intussusception patients in each group was 1223, 1576, and 624, and the incidence rates were 7.85, 11.30, and 4.19 per 100,000 person-half-years (control group 1, control group 2, study group, respectively, *p* < 0.05). No differences in terms of the time from the ED visit to ultrasound, radiological reduction and/or surgical reduction were noted between the study group and the control groups. Conclusions: In Korea, the COVID-19 pandemic did not significantly affect the ED treatment process or the results of patients with intussusception.

## 1. Introduction

The coronavirus disease 2019 (COVID-19) pandemic has had a major impact on societies, lives, economies, and healthcare systems around the world, not only in 2020 but also to date. Globally, several countries have responded to this pandemic with lockdown strategies, including social distancing, school closures, or telecommuting for nonessential workers. The response in South Korea was to reduce the possibility of infection through large-scale testing, contact tracing, and mandated self-isolation in the early phase of the outbreak; consequently, compared to other countries, South Korea has coped relatively well with the COVID-19 epidemic [1]. There have been several reports of a decrease in the number of pediatric patients visiting emergency departments (EDs) during the COVID-19 pandemic [2,3]. In Korea, for instance, the number of pediatric patients visiting EDs for both trauma and medical diseases has decreased [4,5]. There may be several reasons for the decrease in ED visits for pediatric patients, such as fear of exposure and risk of infection, a decrease in the prevalence of infectious disease itself due to infection prevention behavior, and a decrease in access to EDs due to each hospital’s infectious disease strategy. A decrease in healthcare use during a pandemic could negatively impact public health. Failure to access life-saving treatments can increase the severity of the disease, causing complications and even death [6]. During the COVID-19 pandemic, changes in the diagnosis and treatment results for abdominal pain requiring surgery, such as acute appendicitis, and even changes in treatment trends, have been actively reported [7,8,9,10,11,12]. On the other hand, there have been few reports of intussusceptions in pediatric patients. In a study using data from one pediatric emergency center in Korea, it was reported that the number of intussusception patients visiting the emergency department decreased at the same time as infectious diseases after the COVID-19 epidemic [13]. In most cases, the cause of intussusception in children is idiopathic; however, it may be caused by lymphoid hyperplasia due to a viral infection [14]. Globally, pediatric intussusception patients with COVID-19 infection have been reported [15,16,17,18]. In this study, we determined whether the COVID-19 pandemic had an effect on trends in the diagnosis and treatment of pediatric patients with intussusception who required radiological reduction (RR) or surgical reduction (SR).

## 2. Materials and Methods

### 2.1. Study Design and Data Source

This study was a retrospective observational study. We used the National Emergency Department Information System (NEDIS) database, an emergency patient information database operated by the Ministry of Health and Welfare. It includes clinical and administrative data for all patients who have visited EDs nationwide in South Korea. The NEDIS database was established in 2003 to evaluate the quality of the emergency medical system and provide evidence for the development of national emergency medical policy. The number of emergency healthcare facilities participating in the registry was 408 (98.8%) of the 413 EDs in 2016 and 403 (100%) of the 403 EDs in 2020. Medical personnel and administrators of emergency medical centers must enter the required data into the NEDIS system. This database records each patient visit to the emergency room, and multiple visits per patient can be recorded individually. The structure and variables of the NEDIS database have been described in detail elsewhere [19,20]. This study was approved by the institutional review boards of Seoul National University Hospital (approval No. SNUH-2012-104-1183. approval date: 28 December 2020)

### 2.2. Study Period

The period between 1 January (Jan) 2020 and 30 June (Jun) 2020, which occurred during a COVID-19 outbreak, was defined as the ‘COVID-19 period’ (study group). The year of 2019 was divided into two time periods: Jan–Jun (control group 1) and July (Jul)–December (Dec) (control group 2). The characteristics of the three groups of patients were compared.

### 2.3. Participant Selection

#### 2.3.1. Inclusion Criteria

Patients with suspected intussusception who were under the age of 18 years with an International Classification of Disease (ICD) code of K561 as the primary or secondary diagnosis at discharge from the ED or hospital were selected from the NEDIS data. Among these patients, those who underwent either RR or SR were defined as true intussusception patients. We handled recurrent intussusceptions after hospital discharge as a new case; if a repeat reduction was performed after recurring during that same hospitalization, this was treated as the same case.

#### 2.3.2. Exclusion Criteria

Participants with missing demographic information or who were missing an ultrasound (US) time, RR time, or SR time were excluded. In addition, patients with a documented time of US, RR or SR prior to their arrival time at the ED were excluded (time error). Patients who did not undergo US, RR, or SR were also excluded.

### 2.4. Measurements

We collected information on the following variables: the patient’s sex, age and symptom duration (hours); date and time of both visit and discharge from the ED; ED length of stay (EDLOS); whether US was performed, and the date and time it was performed; whether RR was performed, and the date and time it was performed; whether SR was performed, and the date and time it was performed; and disposition.

### 2.5. Outcomes

The primary outcome was the difference between the time from the patient’s ED visit to US examination and from the ED visit to RR and/or SR in the study group, compared to the control groups. The secondary outcomes included the differences in the duration from symptom onset and arrival to the ED, the proportion of patients with intussusception who visited the ED, and the rate of US and/or RR and/or SR between the study group and the control groups.

### 2.6. Statistical Analysis

Categorical variables were assessed with Fisher’s exact test and Pearson’s chi-squared test with post hoc corrections. After confirming whether the continuous variables had a normal distribution, we analyzed the continuous variables using analysis of variance (ANOVA) with a post hoc test for comparisons between the three groups. A *p* value less than 0.05 was considered statistically significant. All statistical analyses were performed using STATA/SE 14.2 software (StataCorp LP, College Station, TX, USA).

## 3. Results

### 3.1. Characteristics of the Patients

**Figure 1:**
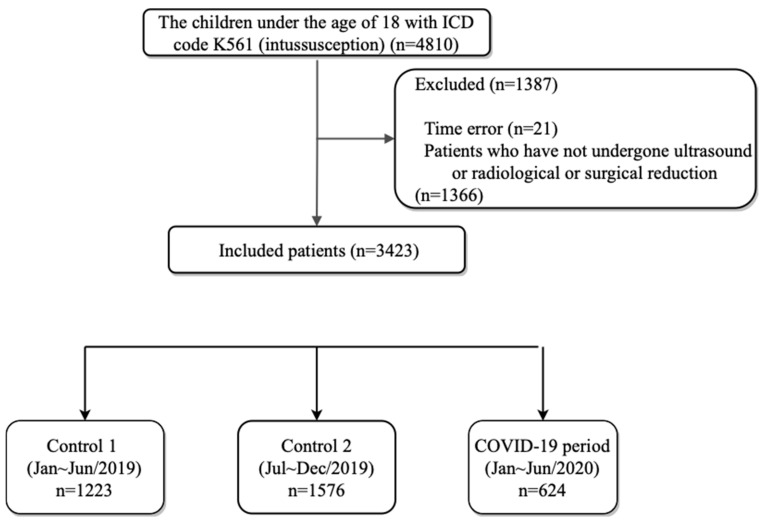
The study flow chart. A total of 4810 patients registered in the NEDIS had intussusception (K561) as the discharge diagnosis. After 1387 patients were excluded, 3423 patients were included in the analysis. There were 1223 patients in control group 1, 1576 patients in control group 2, and 624 patients in the COVID-19 period (study group). (Source: NEDIS: the National Emergency Department Information System.)

The total number of patients visiting EDs was 4,380,894 from Jan–Jun 2019; 4,654,051 from Jul–Dec 2019; and 3,521,808 from Jan–Jun 2020. The number of pediatric patients was 905,529, 929,516, and 524,283 for these time periods, respectively. Overall, from Jan–Jun 2020, the total number of pediatric patients decreased by 42.1% (905,529 => 524,283) compared to that for the same period in 2019. A total of 4810 patients registered in the NEDIS had intussusception (K561) as the discharge diagnosis. Among these, 21 patients who were recorded as having undergone US, RR, or SR before arriving at the ED (time error) were excluded, and 1366 patients who had not undergone US, RR, or SR were also excluded. As a result, 3423 patients were included in the analysis. A total of 1223 (0.14% (1223/905,529)) patients visited the ED from Jan–Jun 2019 (control group 1), and 1576 (0.17% (1576/929,516)) patients visited the ED from Jul–Dec 2019 (control group 2). The number of patients who visited the ED from Jan–Jun 2020, which served as the study group (COVID-19 period), was 624 (0.12% (624/524,283)). This number was significantly reduced compared to that for the same period and the second half of 2019 (Figure 1).

There were 679 (55.52%), 959 (60.85%), and 347 (53.80%) patients with true intussusceptions in control group 1, control group 2, and the study group, respectively. The incidence rates were 7.85, 11.30, and 4.19 per 100,000 person-half-years, respectively. These values were significantly lower in the study group than in control groups 1 and 2 (*p* < 0.05) (Table 1).

The average age of the patients who visited during the COVID-19 period (study group) was 1.95 (±1.87), which was not different from that in the first half of 2019 (1.79 ± 1.54) (*p* = 0.073) but was slightly higher than that in the second half of 2019 (1.77 ± 1.41) (*p* = 0.031). Most of the patients were younger than 6 years of age (98.53, 98.92, and 97.44% for control group 1, control group 2, and the study group, respectively). There were more boys than girls in all three groups (Table 1).

### 3.2. Primary Outcomes

No differences in terms of the time from the ED visit to the time that US was performed and the time from the ED visit to when SR was performed were noted among the groups (*p* = 0.575 and 0.904, respectively) (Table 1). The time from ED arrival to RR was slightly longer in control group 1 than in control group 2 (3.04 ± 6.38 vs. 2.41 ± 2.87, 95% confidence interval (CI): −1.1762 −0.0830), but no difference was noted between the study group and the control groups (Table 1 and Table 2).

### 3.3. Secondary Outcomes

**Figure 2:**
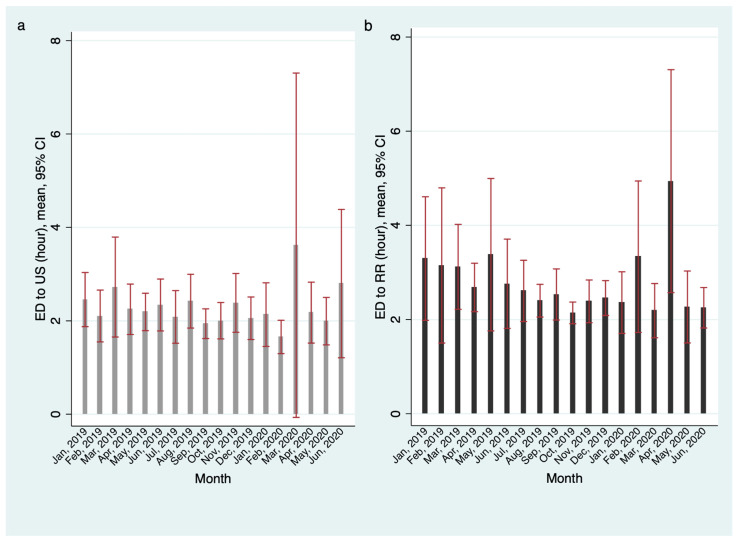
The time interval from arrival at the ED to the time of US and the time from arrival at the ED to RR according to the month of presentation. Monthly, the time interval from arrival at the ED to the time of US (**a**) and the time from arrival at the ED to the time of RR (**b**) did not show a specific trend. ED: emergency department. US: ultrasound. RR: radiological reduction.

No differences in the time from symptom onset to ED admission (symptom duration) or the duration of stay in the ED (EDLOS) were noted among the three groups (*p* = 0.931 and 0.928, respectively) (Table 1). The proportion of patients who underwent US and SR was the same among the three groups, and RR was performed slightly more frequently in control 2 group than in control group 1 and the study group (*p* = 0.001) (Table 1). Figure 2 shows the time from arrival at the ED until US and the time from arrival at the ED to RR according to the month of presentation (Figure 2).

## 4. Discussion

To the best of our knowledge, this study is the first to analyze national data and to show that no problems were encountered in the treatment of pediatric intussusception patients in Korea during the COVID-19 pandemic era. This study shows that the total number of ED visits decreased compared to that before the COVID-19 pandemic, and the number has decreased in pediatric patients at a higher rate than that noted in adults. Additionally, the incidence of pediatric intussusception patients has decreased. However, there were no significant differences in the rate of US, RR, SR, or the time taken for each examination or treatment, and these examinations and treatments were performed for suspected intussusception between the pre-COVID and COVID-19 periods.

Intussusception is the most common cause of small bowel obstruction in children, and mesenteric lymphoid hyperplasia due to viral infection is the main cause. Approximately 67% of cases occur in children under 1 year of age, and most involve children under 6 years of age. In older age groups, pathologic lead points are often associated with intussusception [21]. Nonoperative reduction, such as with air or other hydrostatic reduction methods, has been the standard treatment in most cases, but surgical treatment is required if the reduction fails with noninvasive methods or if complications, such as peritonitis or perforation, occur [21]. Because the purpose of this study was to evaluate whether the COVID-19 pandemic affected the evaluation and treatment process of patients with intussusception in the pediatric ED, all patients under the age of 18 years were included in the analysis.

Pak et al. also reported that the application of appropriate emergency care policies during the COVID-19 pandemic in Korea improved the safety of all emergency patients and reduced hospital mortality [22]. On the other hand, the temporary ED closures because of unpredicted COVID-19 exposure resulted in an increase in in-hospital mortality rates of emergency patients [7]. Therefore, it is very important to properly apply emergency care policies to maintain access to emergency care and prevent unexpected exposure to COVID-19-infected patients. Despite the shortage of resources and manpower due to the COVID-19 pandemic, the total numbers of patients and intussusception patients visiting the ED have decreased. In addition, the application of appropriate emergency care policies seems to have made it possible to evaluate and manage pediatric patients with intussusception in Korea in a timely manner. 

During the COVID pandemic, there have been reports and concerns that the number of ED visits has decreased, and even that the number of patients with serious illnesses has decreased [23,24]. In Korea, there have been reports of a decrease in ED visits by acute myocardial infarction patients or pediatric emergency patients [5,6,19]. Our study also showed that the total number of pediatric visits to EDs and patients with suspected and true intussusception also decreased. Seo S et al. also reported a 3-percentage-point reduction in pediatric intussusception patients in 2020, compared to the pre-COVID-19 pandemic period, in an analysis based on data from three hospitals in Tokyo, Japan [25]. There are two possible reasons for the decrease in the number of pediatric intussusception patients visiting the emergency department. The first could be a decrease in the number of viral infections that cause intussusception, and the second possibility was that the number of emergency room visits had decreased due to concerns about exposure to COVID-19 or the lockdown. Most of the causes of intussusception in children are idiopathic. However, lymphoid hyperplasia caused by a viral infection is often the cause, and the causative viruses are adenovirus, human herpes virus, and Epstein–Barr virus [14]. As infection prevention practices are emphasized and recommended during the COVID-19 pandemic, it could be presumed that the incidence of intussusception has also decreased because other viral infections in children have decreased. This interpretation is supported by a study by Park et al., who reported that a decrease in ED visits for communicable diseases was more pronounced than a decrease in visits for noncommunicable diseases, and the former was significantly correlated with the decrease in visits of intussusception [13]. Children with intussusception may not have visited the ED because their parents were reluctant to visit the hospital. However, these data are national data. In Korea, when a pediatric patient is suspected of having intussusception, other private clinics or secondary hospitals refer the patient to the ED in tertiary centers. In addition, if they had intussusception and did not visit the ED, there was a possibility that they would be admitted to the EDs late due to complications caused by intussusception. However, in this study, there were no significant differences in the time from symptom onset to the time that the patient visited the ED, or in the rate of surgery required, compared to those noted before the COVID-19 pandemic. Therefore, it is difficult to assume that patients with intussusception were inappropriately late on arrival to the ED or did not visit an ED. As a result, it would be reasonable to assume that the incidence of intussusception itself has decreased.

There have been some reports of children with intussusception related to COVID-19 infection, which raised awareness that COVID-19 may be accompanied by intussusception [15,16,17,18]. In Korea, the cumulative number of people under the age of 19 years infected with COVID-19 was 71,923 as of 30 November 2021, and there was only one case of mortality [26]. Compared to other countries, Korea has thus far not had a high rate of COVID-19 infection among children. Therefore, it is not yet appropriate to analyze whether the incidence of intussusception increased after the start of the COVID-19 pandemic.

This study has some limitations. The first limitation is that the list of study patients was extracted with the diagnostic code registered in the NEDIS database. For various reasons, an incorrect diagnostic code or a diagnostic code used in the past may have been reported. As a result, the list of participants included patients who were discharged without US or any form of reduction. This is considered to be a limitation of such a large public database, and these patients had to be excluded and analyzed as patients without intussusception. However, this study is meaningful because it is the result of analyzing the best-quality emergency medical system database in Korea, given that the NEDIS is a well-managed and validated database. Second, patients who were discharged from the hospital who underwent US but who did not have RR or SR were included. These may include patients with nonileocolic intussusception or spontaneously reduced intussusception. Alternatively, there may be cases in which intussusception was not diagnosed upon US examination, but the K561 diagnosis code was entered for the claim or a reduction was not required, such as was noted in cases of small bowel intussusception. Given that these patients were also suspected to have intussusception and underwent US, they were defined as having suspected intussusception and were included in the analysis. Third, because the data matched by the EMR of each hospital are transmitted to the NEDIS, there are cases where an error occurred regarding the time point. In a few patients, US, RR or SR times were recorded prior to the time they visited the ED. Their data were also excluded. Because there are small numbers of such patients in the total data, these patients should not have had a significant impact on the results.

## 5. Conclusions

In Korea, compared to those before the COVID-19 pandemic, the total number of pediatric patient visits to EDs and the number of patients with intussusception decreased, and the COVID-19 pandemic did not significantly affect the ED treatment process or the results of patients with intussusception. Additional research is needed on the status of pediatric patients visiting the emergency room with other diseases unrelated to infectious diseases, and on establishing a plan to continuously respond to the ongoing pandemic according to the research results. In the future, we will have to think about how to improve the pediatric emergency medical system in case another pandemic occurs.

## Figures and Tables

**Table 1:** Patient characteristics.

	Control Group 1 (Jan~Jun, 2019)		Control Group 2 (Jul~Dec, 2019)		Study Group (Jan~Jun, 2020)		Total		*p*
Intussusception suspected	1223		1576		624		3423		
True intussusception, *n* (%)	679	(55.52%)	959	(60.85%)	347	(53.80%)	1985	(57.99%)	0.007
True intussusception, n/100,000 (person-half-year)	7.85		11.30		4.19		7.77		<0.05
Age, mean ± SD	1.79	±1.54	1.77	±1.41	1.95	±1.87	1.81	±1.55	0.034
Patients younger than 6 years old, *n* (%)	1205	(98.53%)	1559	(98.92%)	608	(97.44%)	3372	(98.51%)	
Male, *n* (%)	771	(63.04%)	998	(63.32%)	394	(63.14%)	2163	(63.19%)	0.988
Symptom duration (hr), mean ± SD	20.21	±31.28	20.17	±42.65	20.85	±43.83	20.31	±39.20	0.931
Intervention									
US, *n* (%)	1087	(88.99%)	1407	(89.28%)	555	(86.05%)	3049	(88.53%)	5
RR, *n* (%)	664	(54.29%)	934	(59.26%)	333	(51.63%)	1931	(56.07%)	0.001
SR, *n* (%)	26	(2.13%)	34	(2.16%)	20	(3.10%)	80	(2.32%)	0.347
The time to intervention									
ED~US (hr), mean ± SD	2.34	±4.39	2.16	±3.92	2.35	±7.13	2.26	±4.83	0.575
ED~RR (hr), mean ± SD	3.04	±6.38	2.41	±2.87	2.79	±4.39	2.69	±4.61	<0.05
ED~SR (hr), mean ± SD	11.61	±23.45	9.23	±21.37	10.11	±13.17	10.22	±20.19	0.904
ED LOS (hr), mean ± SD	4.62	±4.15	4.67	±4.10	4.68	±4.13	4.65	±4.12	0.928

US: ultrasound. RR: radiological reduction. SR: surgical reduction. ED: emergency department. LOS: length of stay.

**Table 2:** Time interval from patient arrival to the ED to the time of US, RR or SR.

	ED to US		ED to RR		ED to SR	
	Difference	95% CI	Difference	95% CI	Difference	95% CI
Control 1 vs. 2	−0.1829	−0.6468~0.2809	−0.6296	−1.1762~−0.0830	−2.3823	−15.0973~10.3327
Control 1 vs. Study	0.0114	−0.5848~0.6077	−0.2501	−0.9703~0.4699	−1.4978	−16.0137~13.0181
Control 2 vs. Study	0.1944	−0.3783~0.7670	0.3794	−0.3038~1.0627	0.8846	−12.8688~14.6379

ED: emergency department. US: ultrasound. RR: radiological reduction. SR: surgical reduction.

## Data Availability

https://www.dropbox.com/home/1.%20Mac_%EB%B3%91%EC%9B%90%EA%B4%80%EB%A0%A8/1.%20%EC%97%B0%EA%B5%AC/%231.%20intu%20outcome%20in%20COVID-era?preview=NEDIS_intu+v3.2+(time+error+delete).xlsx.
